# Quality assurance of electron and photon beam energy using the BQ‐Check phantom

**DOI:** 10.1120/jacmp.v12i2.3366

**Published:** 2011-02-01

**Authors:** Richard J. Speight, Ashraf Esmail, Steve J. Weston

**Affiliations:** ^1^ Medical Physics and Engineering St. James's University Hospital, Leeds Teaching Hospitals Trustz Leeds LS9 7TF England

**Keywords:** linear accelerators, radiotherapy, commissioning, beam quality, energy‐equivalent parameters

## Abstract

The BQ‐CHECK phantom (PTW Freiburg, Germany) has been designed to be used with a 2D ion chamber array to facilitate the quality assurance (QA) of electron and photon beam qualities (BQ). The BQ‐CHECK phantom has three wedges covering the diagonal axes of the beam: two opposed aluminum wedges used to measure electron energy and a single copper wedge used to measure photon energy. The purpose of this work was to assess the suitability of the BQ‐CHECK phantom for use in a routine QA program.

A range of percentage depth dose (PDD) curves for two photon beams and four electron beams were measured using a MP3 plotting tank (PTW Freiburg). These beams were used to irradiate a STARCHECK array (PTW Freiburg) with and without the BQ‐CHECK phantom on top of the array. For photons, the ratio of the signals from two chambers underneath the copper wedge was used as an effective TPR measurement $\left({\text{TPR}}_{\text{eff}}\right)$ and, for electrons, the full width at half maximum of the profile $\left(E_{\text{FWHM}}\right)$ underneath the aluminum wedges was used as an electron energy constancy measurement. PDD measurements were compared with ${\text{TPR}}_{\text{eff}}$ and $E_{\text{FWHM}}$ to assess the sensitivity of the BQ‐CHECK phantom.

The clinical tolerances of ${\text{TPR}}_{\text{eff}}$ were determined for 6 MV (0.634–0.649), and 10MV (0.683–0.692). For electrons, the clinical tolerances of $E_{\text{FWHM}}$ were determined for 6 MeV (94.8–103.4 mm), 8 MeV (105.5–114.0 mm), 10 MeV (125.4–133.9 mm) and 12 MeV (138.8–147.3 mm).

Electron and photon energy metrics are presented which demonstrate that the BQ‐CHECK phantom could be used to form part of an efficient routine monthly QA program. Acceptable beam quality limits for various nominal beam energies were established and at these limits, modified profiles were acquired using the STARCHECK array. From the modified profiles, $E_{\text{FWHM}}$ and ${\text{TPR}}_{\text{eff}}$ were determined for the electron and photon beams, respectively. It was demonstrated that both $E_{\text{FWHM}}$ and the ${\text{TPR}}_{\text{eff}}$ have a linear relationship with conventional beam quality metrics.

PACS numbers: 87.56.bd, 87.56.‐v

## I. INTRODUCTION

It is recommended that quality assurance (QA) of the beam energy of radiotherapy treatment machines is carried out at regular intervals, either weekly^(1)^ or monthly.^(^ ^2^ ^–^ ^3^ ^)^ The standard for beam energy characterization is the measurement of depth ionization curves or depth dose curves in a water phantom using a suitable detector,^(4)^ and usually a measurement such as dose at depth is taken as the metric. However, such measurements are time‐consuming and impractical on a regular basis in the clinical environment, and often sampling of the beam at various depths in a solid phantom is used as the QA measurement to ensure that the beam energy is consistent with commissioning data.^(3)^

Measuring beam energy can be made easier by producing pseudodepth‐ionization curves using wedged phantoms aligned with the axis of the beam. Measurements taken from the profile underneath the wedge can be correlated with the beam energy.^(^ ^5^ ^–^ ^7^ ^)^ Wells et al.^(8)^ introduced a double wedge technique for measuring electron beam energy that is invariant to phantom alignment in the wedge direction, and had a similar sensitivity to water tank measurements for electron energies between 6 and 20 MeV. They used a phantom with two PMMA wedges attached to a diode array to produce profiles at a fixed source‐to‐surface distance (SSD) and field size. From these electron profiles, the half width at half maximum (which they called ${\text{EC}}_{50}$) was measured and shown to be proportional to $R_{50}$ (the depth of 50% dose along the central axis) for the electron energies tested.

For photons, measuring the beam energy is more complex as it is dependent on the maximum electron energy, the electron energy distribution, and both the target material and thickness. Beam energy can be evaluated using the doses measured at two depths in either water or solid phantom with constant SSD; or constant source‐to‐chamber distance (SCD). The normalized charge readings from the ionization chambers under a wedge is effectively a depth dose curve (ignoring off‐axis beam hardening effects) from which an effective tissue phantom ratio $\left({\text{TPR}}_{\text{eff}}\right)$ can be generated. This effective TPR can be used as an evaluation of the beam energy. Chofor et al.^(9)^ suggested using values at equivalent water thicknesses of 30 and 15 cm to get ${\text{TPR}}_{15/30}$, which is sensitive to energy changes.

**Figure 1:**
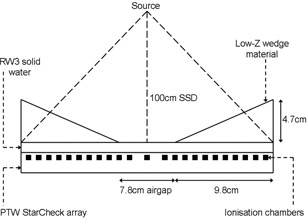
A schematic diagram of the experimental setup and dimensions of the PTW BQ‐CHECK phantom.

The BQ‐CHECK phantom has been developed to be used in conjunction with the STARCHECK or 2D‐ARRAY seven29 ionization chamber arrays (both PTW Freiburg) for the QA of electron and photon beam energy. The BQ‐CHECK phantom is 300×300×47 mm in size with wedges at three of the diagonal corners: two opposing aluminum wedges and a single copper wedge. Using an array and the BQ‐CHECK phantom, the principal axis symmetry and flatness, as well as the beam energy, can be assessed simultaneously. The arrays have inherent buildup of approximately 7 mm;^(10)^ therefore, solid water sheets must be placed on top of the array to provide sufficient buildup (Fig. 1).

## II. MATERIALS AND METHODS

Synergy platform linear accelerators (Elekta Oncology Systems, Crawley, UK) were used in the current work, with 6 and 10 MV photon beams and 6, 8, 10, and 12 MeV electron beams.

The current definitive parameters for clinical QA of electron beam and photon energies at SJUH are the depth of 80% dose $\left(R_{80,D}\right)$ and percentage depth dose at 10 cm depth $\left({\text{PDD}}_{10}\right)$, respectively. Both are measured in a plotting tank (MP1‐S or MP3, PTW Freiburg) with a Markus chamber (Type 23343, PTW Freiburg), and data is collected and analyzed using the MEPHYSTO software (PTW Freiburg). The clinical tolerances on the nominal $R_{80,D}$ and ${\text{PDD}}_{10}$ values are ± 2 mm and ± 1%, respectively. For electrons, the measurements were taken with a 200×200 mm electron applicator and standard end frame. The height of the plotting tank was adjusted until the SSD was 950 mm, which is the standard clinical setup used at SJUH. For photons, the measurements were taken with a 100×100 mm field size at 1000 mm SSD.

To establish acceptable tolerance limits for the BQ‐CHECK phantom measurements, the machine parameters (e.g., bending magnet current and gun current) required to modify the beam energy were determined, and the corresponding $R_{80,D}$ and ${\text{PDD}}_{10}$ values were found from depth doses curves measured using the plotting tank. The range of beam energies was chosen so that it encompassed the clinical tolerances of the nominal beams.

The plotting tank was removed and the SC universal gantry mount (PTW Freiburg) was attached to the accessory ring on the linear accelerator. The STARCHECK array was inserted into the array holder and the configuration was adjusted so that the SSD was 1000 mm. For electron beam and photon measurements, 5 and 20 mm, respectively, of RW3 solid water was placed on top of the STARCHECK array. For each beam configuration, which was determined by modifying the machine parameters, one measurement was taken without the BQ‐CHECK phantom and one measurement was taken with the BQ‐CHECK phantom in place. For the photons, the field size used to irradiate the STARCHECK array was 240×240 mm. Profiles were measured with the MultiCheck application (PTW Freiburg).

The electron beam energy was assessed from the profile across the two opposing aluminum wedges. The quotient of the profiles both with and without the BQ‐CHECK phantom was produced to account for dose differences from the central axis, and normalized on its central axis. The energy‐equivalent parameter was chosen as the full width at half maximum of the modified electron profiles $\left(E_{\text{FWHM}}\right)$

For photons, beam energy was assessed from the profile of the single copper wedge. Again the quotient of the profiles both with and without the BQ‐CHECK phantom was produced and normalized on its central axis. The energy‐equivalent parameter was chosen as an effective TPR $\left({\text{TPR}}_{\text{eff}}\right)$, which was defined as the normalized signal from the 36th ionization chamber from the central axis divided by the normalized signal from the 21st ionization chamber from the central axis. The 21st and 36th chambers were chosen because their normalized signals were equivalent to the 6 MV PDD values at 100 and 200 mm depths, respectively, given the water equivalence of the metal above their positions.

## III. RESULTS

### A. Electron results

**Figure 2:**
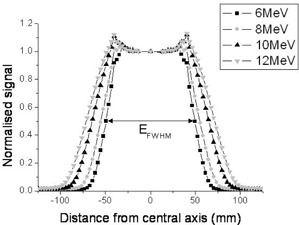
Normalized signal profiles taken through the two opposing aluminum wedges in the BQ‐CHECK phantom. The profiles for all four nominal energies are shown, along with the measurement of $E_{\text{FWHM}}$ for the 6 MeV beam.

**Figure 3:**
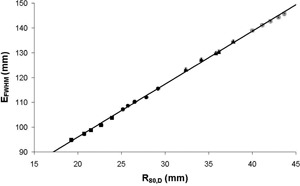
$E_{\text{FWHM}}$ as a function of $R_{80,D}$ for all energies. Results were taken at the following nominal electron energies: 6 MeV (■), 8 MeV(●), 10 MeV(▲), and 12 MeV(*).

The electron beam energy was varied so that five different beams with known values of $R_{80,D}$ could be produced for each of the four nominal electron energies. Modified profiles were measured at all known beam energies. The normalized profiles for each of the nominal electron beam energies are shown in Fig. 2. $E_{\text{FWHM}}$ was measured for all the profiles and has been plotted against $R_{80,D}$ (Fig. 3). The tolerance for $R_{80,D}$ for each nominal energy is ± 2 mm and, from Fig. 3, the tolerance limits for $E_{\text{FWHM}}$ were assessed (Table 1).

**Table 1:** Acceptable $E_{\text{FWHM}}$ values for various nominal electron energies for the specific linear accelerator used.

*Nominal Electron Energy (MeV)*	*Acceptable Range of* $E_{\text{FWHM}}$ *(mm)*
6	94.8–103.4
8	105.5–114.0
10	125.4–133.9
12	138.8–147.3

### B. Photon results

**Figure 4:**
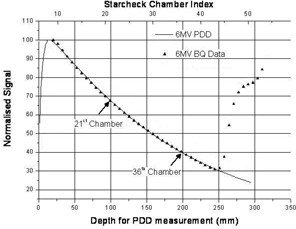
PDD curve measured at 6 MV and the normalized signal profiles taken under the single copper wedge of the BQ‐CHECK phantom; the positions of the 21st and 36th ionization chambers are displayed. Points measured beyond the 44th chamber are at the maximum height of the wedge and should not be considered.

**Figure 5:**
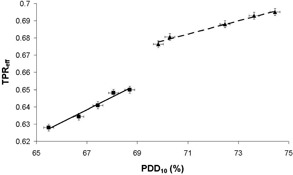
${\text{TPR}}_{\text{eff}}$ as a function of ${\text{PDD}}_{10}$ for all energies. Results were taken at the following nominal photon energies: 6 MV (■) and 10 MV (▲). Lines of best fit were added separately for each nominal energy.

The beam energy was varied so that five different beams with known values of ${\text{PDD}}_{10}$ could be produced for each of the two nominal photon energies. Modified profiles were measured at all known beam energies. Aligning $d_{\text{max}}$ from the modified profile and the PDD curve and scaling the STARCHECK chamber index shows the relation between the attenuation of the depth dose in water against the attenuation of the profile by the copper wedge. This has been done for one photon energy of 6 MV (see Fig. 4). This figure demonstrates that the ratio of the signals from the 36th and 21st chamber from the central axis is equivalent to the ratio between ${\text{PDD}}_{20}$ and ${\text{PDD}}_{10}$ for 6 MV. For this reason, the authors have chosen the ratio of the signals from the 36th and 21st chamber from the central axis, ${\text{TPR}}_{\text{eff}}$, as the QA parameter to be used as a measure of photon energy constancy. For other beam qualities, this equivalence does not strictly apply; however, the use of the signals from these chambers is still justified, as demonstrated by Fig. 5.

${\text{TPR}}_{\text{eff}}$ was plotted as a function of ${\text{PDD}}_{10}$ for all profiles (see Fig. 5). It can be seen that, for each nominal energy, ${\text{PDD}}_{10}$ is proportional to ${\text{TPR}}_{\text{eff}}$. Thus, if ${\text{TPR}}_{\text{eff}}$ was measured during the monthly QA, ${\text{PDD}}_{10}$ could be determined. The tolerance for ${\text{PDD}}_{10}$ for each nominal energy is ± 1% and hence, from Fig. 5, the tolerance limits for ${\text{TPR}}_{\text{eff}}$ were determined (Table 2).

**Table 2:** Acceptable ${\text{TPR}}_{\text{eff}}$ values for various nominal photon energies for the specific linear accelerator used.

*Nominal Electron Energy (MV)*	*Acceptable Range of* ${\text{TPR}}_{\text{eff}}$
6	0.634–0.649
10	0.683–0.692

## IV. DISCUSSION

### A. Electron measurements

Figure 3 demonstrates the linear relationship between the standard energy characterization parameter $R_{80,D}$ and the measured parameter $E_{\text{FWHM}}$. The error in the $E_{\text{FWHM}}$ measurement was estimated from a series of 10 repeated measurements to be 1.4 mm (2SD). This correlates to an accuracy of 0.7 mm in the determination of $R_{80,D}$. When compared to the tolerance value of 2 mm, this indicates that the BQ‐CHECK phantom is an appropriate tool to use for the routine constancy measurement of electron beam energy.

### B. Photon measurements

Figure 5 shows that the relationship between ${\text{TPR}}_{\text{eff}}$ and ${\text{PDD}}_{10}$ is different for different nominal beam energies. This is due to the effect of the different filter combinations on the profile of the beam. The gradient of the graph indicates the sensitivity of ${\text{TPR}}_{\text{eff}}$ in the quality assurance of the photon beam energy. The error in ${\text{TPR}}_{\text{eff}}$ was estimated from a series of 10 repeated measurements to be 0.002 (2SD). This correlates to an accuracy in ${\text{PDD}}_{10}$ of 0.3% and 0.5% for 6 and 10 MV, respectively. When compared to a tolerance value of 1.0%, this indicates that the BQ‐CHECK phantom may be suitable for photon energy QA, but it should be assessed for each nominal beam energy and filter combination.

## V. CONCLUSIONS

It has been shown that the BQ‐CHECK phantom combined with the STARCHECK array can be used in the quality assurance of photon and electron beam energy. Two different parameters, $E_{\text{FWHM}}$ and ${\text{TPR}}_{\text{eff}}$, have beam evaluated against electron and photon beam energy, respectively. Both of these parameters are linear with the conventional beam energy quality assurance parameters and, as a result, can be used to establish the constancy of electron and photon beam energy. Acceptable ranges of these energy‐equivalent parameters have been established for implementation in a QA program.

## ACKNOWLEDGMENTS

The authors thank Rudiger Lauk and Tobias Kremp of PTW Freiburg and Gavin Cranmer‐Sargison from the Saskatoon Cancer Centre for their help and assistance with this work.
